# Comparability of Archived Aging-Stage and Retest Records from Miniature Temperature–Pressure Sensors: Rank, Concordance, and Response Shape

**DOI:** 10.3390/s26175650

**Published:** 2026-09-05

**Authors:** Zhiyuan Zhou, Xuecheng Dong, Liangzhu Yan, Lingyun Wang

**Affiliations:** 1 College of Energy (College of Modern Shale Gas Industry), Chengdu University of Technology, No. 1 Erxianqiao East 3rd Road, Chenghua District, Chengdu 610059, China; 2 State Key Laboratory of Oil and Gas Reservoir Geology and Exploitation, Chengdu University of Technology, No. 1 Erxianqiao East 3rd Road, Chenghua District, Chengdu 610059, China; 3 No. 10 Oil Production Plant of PetroChina Changqing Oilfield Company, Qingyang 745000, China

**Keywords:** miniature temperature–pressure sensor, retest comparability, rank retention, concordance, temperature support, response profile

## Abstract

Archived sensor records often lack synchronized reference pressure, making it necessary to distinguish retained device ordering from numerical agreement and response-shape reproduction. Aging-stage and first-retest records from 22 miniature temperature–pressure sensors were evaluated using marginal features, Spearman rank correlation, Lin’s concordance correlation coefficient (CCC), device-level bootstrap intervals, and 56 equally weighted common-temperature bins. A disjoint 16-device same-mode cohort tested recurrence of marginal-feature ordering. The analysis demonstrates the following. The fifth percentile retained device order (0.886) and identity-line agreement (CCC 0.968); standard deviation retained order (0.648) without agreement (CCC 0.032), which the decomposition attributes to systematic displacement (accuracy factor 0.077), whereas the 95th percentile (CCC 0.379) is precision-limited (Pearson *r* 0.510). Common-temperature alignment reversed the raw-median difference for every device. Relative temperature-slope retention was negligible (rank correlation 0.013), and complete centered-profile correlation was heterogeneous (median 0.356). Lower-tail, upper-tail, and dispersion ordering recurred in the disjoint cohort. The article proposes a multilevel retention signature, an ordinal pattern read across evidence levels, together with candidate retest endpoints. Calibration accuracy, sensor life, sensitivity degradation, retention of metrological characteristics, and causal aging lie beyond this evidence.

## 1. Introduction

Miniature temperature–pressure sensors extend measurement from remote wellbore locations toward the process that must be interpreted. Fiber Bragg grating systems have enabled casing-pressure acquisition under downhole constraints [1], while carbon-coated, bellows-packaged optical sensors have provided simultaneous pressure and temperature records in oil wells [2]. Structural modifications such as side-hole fiber Bragg gratings further show that sensor geometry can change pressure sensitivity without removing the need for temperature measurement [3]. Silicon-based Fabry–Pérot elements extend the same coupled measurement into high-temperature service [4]. Near-bit instruments move this coupled measurement still closer to the drilling action [5]. The resulting record is valuable only if its meaning remains traceable when a device is packaged, exposed, stored, and retested.

Moving the measurement point closer to the bit also makes the complete acquisition chain more consequential. Microelectromechanical system (MEMS)-based downhole measurers integrate several sensing functions within a restricted package [6], and compact micro-measurers extend this integration into measurement-while-drilling workflows [7]. Sustained operation at elevated temperature additionally requires distributed thermal management of the downhole electronics themselves [8]. Reviews of resource-exploration sensors identify packaging and environmental adaptation as determinants of performance rather than secondary implementation details [9]. Probe-type intelligent-bit tests likewise require comparison with other bottom-hole and logging records before the measured parameters can be interpreted [10]. Multiparameter sensing under extreme conditions is therefore a central enabling element of intelligent drill bits [11]. These applications create a common metrological question: when a sensor is tested again, which part of its earlier output structure remains comparable?

That question is not answered by a batch mean or a single correlation coefficient. Nominally similar piezoresistive pressure sensors can retain individual differences in sensitivity, nonlinearity, and temperature behavior during long-term tests [12]. Such differences can originate from sensitive-structure design, fabrication, and packaging [13]. Packaging operations can also move zero output much more than sensitivity, hysteresis, or repeatability, demonstrating that response components need not change together [14]. Controlled thermal cycling further shows that individual package elements contribute unequally to hysteresis and may stabilize at different rates [15]. A monotone stage-wise shift may therefore preserve device ranks while moving values away from the identity line; conversely, similar central values may conceal changes in dispersion or response shape. Retest comparability must distinguish these outcomes rather than collapse them into a binary stable/unstable label.

Established practice offers four families of solutions to the question of whether a device’s characteristics have been retained, and it is useful to state what each of them requires. The first is controlled long-term stability testing, in which a device is monitored against a reference over an extended interval: a resonant pressure microsensor evaluated under defined pressure and temperature tests achieved a reported zero-point stability error of 0.01% full scale over five months [16], and an implanted pressure sensor tracked against a reference over months located the drift in the package [17]. The second is accelerated aging under a defined exposure, which compresses the time axis by applying a known thermal or electrical stress and comparing characteristics measured before and after it [18]; extended thermal aging of a bandgap voltage reference, for example, has been traced to oxidation of the molding compound [19]. The third is periodic recalibration on a schedule, for which formal methods exist to set and revise the calibration interval from a device’s own history [20], resting in turn on an unbroken traceable calibration chain [21]. The fourth is statistical method comparison, which asks whether two sets of measurements agree, a stricter condition than correlation: concordance and limits-of-agreement procedures are standard for that purpose [22], and both their reporting conventions [23] and the conditions under which a limits-of-agreement analysis is admissible at all [24] have been examined in detail.

Each of these families presupposes something that an incomplete archive does not contain. The first three require a traceable reference, a documented exposure, or a known elapsed time. The fourth requires that the two measurement sets be replicates obtained under a single controlled protocol, which two archived record groups of undocumented procedure are not. A parallel difficulty arises wherever condition assessment is attempted from operational records: multisensor arrays diagnosing thermal faults in power cables [25] and models issuing early warnings from wind-turbine gearbox oil temperature [26] both depend on continuous, densely instrumented monitoring streams, whereas an archive of two isolated test campaigns offers neither continuity nor an operational baseline. Calibration frameworks in other sensing modalities meet related difficulties by making the error model explicit and regularizing the estimate [27]. For records that meet none of these preconditions, a framework is needed that states which comparisons the records still support.

Temperature introduces a second source of ambiguity because it is both an environmental input and an index of the test path. Zero-point output carries a temperature-dependent component of its own, which has to be characterized on a controlled thermal path before it can be corrected [28], and wide-range digital compensation studies likewise characterize temperature-dependent offset and sensitivity explicitly before applying a correction [29]. Consequently, unequal temperature coverage or dwell can change a marginal output distribution without identifying a change at matched temperature. Proper calibration resolves this ambiguity by coupling the sensor to a reference pressure source and controlled thermometry; compensation studies similarly require synchronized pressure and temperature standards [30]. Dynamic thermal lag can still limit a relation obtained under static conditions [31]. Historical records that omit those controls can support a narrower but useful question about output comparability, provided the claim is not converted into calibration accuracy or causal degradation.

Because calibration accuracy cannot be reconstructed from these records, the tractable question is which device-level structures remain comparable. Three questions organize the study. First, do marginal output features preserve device order and proximity to the identity line simultaneously? Second, does restricting both stages to common temperature support change the inferred stage difference or the ordering of temperature-dependent features? Third, does a favorable scalar result extend to the complete response profile and recur in a disjoint repeated-record cohort? To answer these questions, the study combines rank, concordance, support-aligned, and functional evidence in one device-level workflow. The analysis shows that ordering can persist without numerical agreement and that the retained temperature domain can reverse the apparent record difference. The contribution is a multilevel comparability framework for incomplete sensor archives, together with an explicit account of the additional measurements required before that framework can be connected to calibration or qualification.

## 2. Materials and Methods

### 2.1. Sensor Records and Pairing Design

The primary cohort comprised 22 miniature temperature–pressure sensors from one laboratory test batch. Each device had one record labeled as an aging record in the source archive and a first subsequent record stored in the calibration directory. Device identifiers provided a one-to-one pairing. Each retained record contained 6143–6144 valid pairs of temperature and pressure-channel output. Across the 22 records, measured temperature extended from 16.125 to 82.8125 °C in the aging-labeled stage and from 11.5625 to 85.0625 °C at first retest.

The aging-stage label identifies the original record group in the source archive and functions as a group name. Defining an exposure would require a documented duration, temperature, and load, so the label is treated as an archive attribute throughout. Section 2.2 sets out the procedure information the archive carries and the evidence boundary that follows from it.

For both primary-stage folders, the first two columns of each comma-separated-value (CSV) file were interpreted as temperature and pressure-channel output. Values were coerced to numeric form, and a row was removed only when either member of the pair was missing or non-numeric. No smoothing, interpolation, outlier deletion, or within-record resampling was applied before feature extraction. Quantiles used linear interpolation, and standard deviation used the n−1 denominator. These choices preserve the archived empirical distributions and make each transformation reproducible from the supplied script.

Consecutive observations within a record were used to estimate a device’s features, but they were not treated as independent inferential replicates. The device was the statistical unit (n=22), so all confidence intervals, tests, and deletion analyses resampled or removed complete devices. Board-level records with a different assembly status were excluded from the aging-to-retest pairing because assembly and procedure effects could otherwise be conflated with the record label.

A distinct same-mode cohort was constructed to assess whether the marginal-feature hierarchy recurred under another pairing rule. Direct instrument exports were screened programmatically. A record was eligible when its mode field ended in 0, it contained at least 1000 valid temperature–output pairs, all decoded temperatures fell within 10–90 °C, and its temperature span was at least 50 °C. Duplicate contents were removed by Secure Hash Algorithm 256-bit (SHA-256) hash. Devices with at least two unique eligible records contributed the first and last filenames under the archive’s chronological naming convention, producing 32 records from 16 devices. All retained exports were mode 0, recorded a 5.0 s sampling period, and carried the same non-rechargeable product-type label. No device identifier overlapped with the 22-device primary cohort. The repeat exports label their output scale as voltage, whereas the primary pressure-channel scale is unconfirmed; consequently, only scale-free rank evidence is compared across cohorts. This disjoint cohort tests recurrence of a repeated-record feature hierarchy, not the same primary devices, an untreated control, or an independent aging experiment.

**Figure 1:**
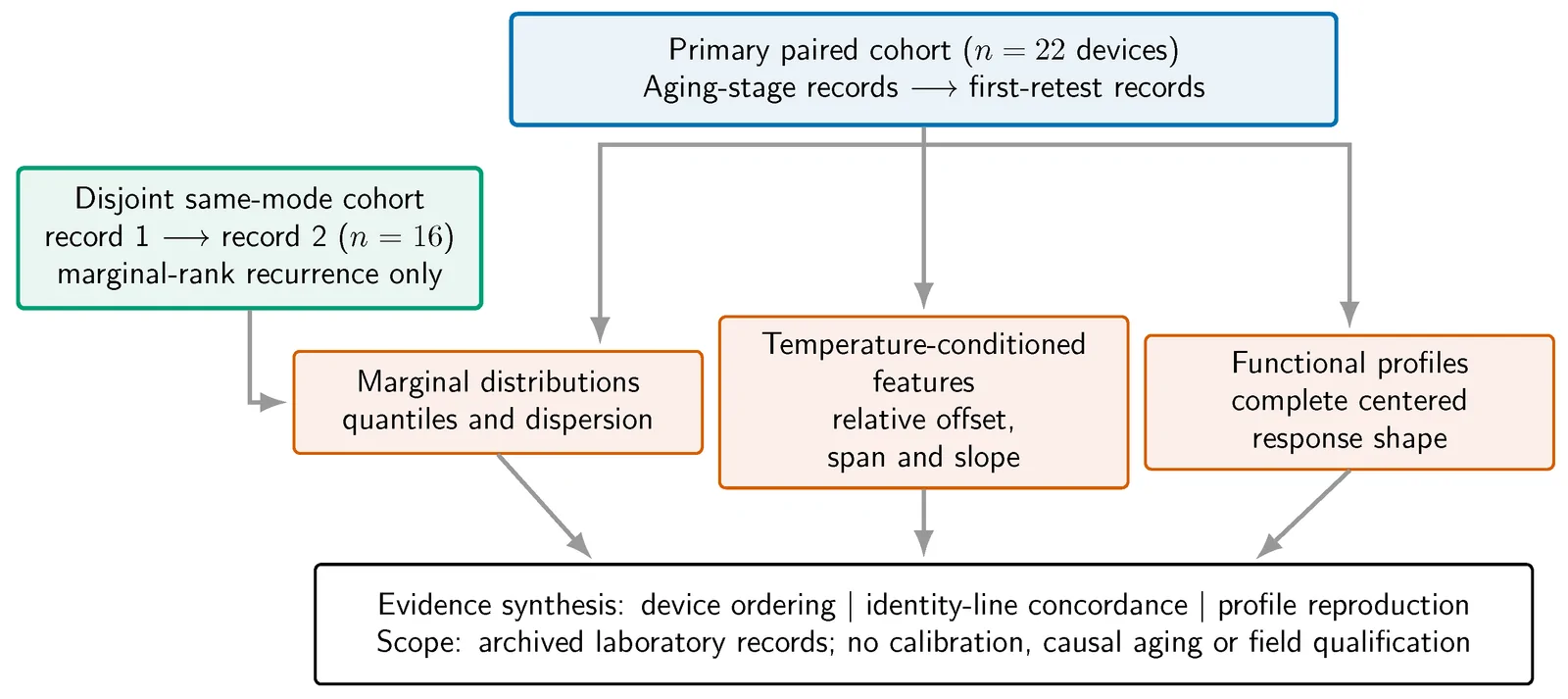
Analytical framework for cross-record feature retention. The primary paired cohort contributes marginal, temperature-conditioned, and functional evidence; the disjoint same-mode cohort tests recurrence only for marginal-feature ranks. The final box defines the claim boundary imposed by the archived records.

The two cohorts have deliberately different evidential roles: the primary pairing supplies all three comparison levels, whereas the disjoint cohort challenges only recurrence of marginal-feature ordering (Figure 1).

### 2.2. Evidence Boundary of the Archived Records

The procedure information carried by the archive was inventoried before any comparison was made, and it defines the scope of the study (Table 1). The records supply paired temperature and pressure-channel sequences with a one-to-one device correspondence, an aging-stage group label, and a partial account of assembly status. Three further groups of information would be needed to convert a cross-record comparison into a statement about device degradation. The first is a defined exposure: a duration, temperature, load, and operating mode for the aging interval, together with the elapsed time before first retest, which together fix a thermal and mechanical dose. The second is a traceable measurement chain: a synchronized reference pressure and a confirmed physical unit for the pressure-channel field, which together place output values on a metrological scale; in their absence, values and differences are reported in the recorded unit. The third is a reconstructable test path: row-level timestamps, the loading program, path direction, and dwell.

The estimand adopted here follows directly from that scope: the persistence of between-device output structure across two archived record groups generated under the laboratory procedures of the time. Accuracy, sensitivity, nonlinearity, hysteresis, repeatability, drift rate, elapsed-time degradation, sensor life, and the retention of metrological characteristics each depend on one of the three groups above, and they are accordingly carried forward as objects for a confirmatory study. The same scope governs the results, discussion, and conclusions below, and Section 4.4 specifies the measurements that would extend it.

### 2.3. Marginal, Temperature-Conditioned, and Functional Features

Let $T_{i,s}$ and $P_{i,s}$ denote the temperature and pressure-channel output sequences for device *i* in stage *s*. Five complementary marginal features were calculated from each complete pressure-output sequence: the 5th, 50th, and 95th percentiles ($Q_{0.05}$, $Q_{0.50}$, and $Q_{0.95}$), the sample standard deviation *S*, and the central 90% span

(1) $$D_{90}=Q_{0.95}-Q_{0.05}.$$

The three quantiles locate the lower tail, center, and upper tail without assuming a parametric distribution. *S* captures total dispersion, whereas $D_{90}$ reduces the influence of the most extreme observations. Because the marginal features retain the original temperature weighting of each record, they are useful for describing archived records but are not temperature-adjusted sensor parameters.

Temperature conditioning compared the stages on shared support rather than on their original dwell-weighted distributions. For bin width *h*, an observation was assigned to center *t* when T∈[t−h/2,t+h/2), with centers anchored at 22 °C. The device–stage conditional output was

(2) $$g_{i,s}\left(t\right)=\mathrm{median}\left\{P_{i,s,k}:T_{i,s,k}\in \left[t-h/2,t+h/2\right)\right\}.$$

The primary analysis used °C. A bin was retained only when every device contributed observations at both stages, giving 56 centers between 22 and 80 °C; centers at 35, 38, and 45 °C were absent and were not interpolated. Subsequent summaries gave each retained center equal influence, so the conditioned estimand differs deliberately from the observation-weighted raw distribution. Presence, rather than a minimum within-bin count, defined the main support. Counts ranged from 1 to 3332 observations per device–bin in the aging-stage records and from 4 to 1802 at retest, with a median of six in both stages. Medians were chosen because the archived traces contain boundary transitions and unequal dwell densities for which the conditional mean would be less robust.

For each device, a leave-one-device-out cohort median was formed at every common bin. The centered profile

(3) $$c_{i,s}\left(t\right)=g_{i,s}\left(t\right)-{\mathrm{median}}_{j\neq i}\left\{g_{j,s}\left(t\right)\right\}$$

expresses the device’s position relative to its peers without allowing the device to contribute to its own reference. Three scalar summaries were derived: the relative offset $A_{i,s}={\mathrm{median}}_{t}c_{i,s}\left(t\right)$; the centered 90% span $W_{i,s}=Q_{0.95,t}\left\{c_{i,s}\left(t\right)\right\}-Q_{0.05,t}\left\{c_{i,s}\left(t\right)\right\}$; and the Theil–Sen slope $\beta_{i,s}$ of $c_{i,s}\left(t\right)$ against bin-center temperature. *A* measures persistent vertical position within a cohort, *W* measures temperature-dependent variation in relative position, and β measures its monotonic component.

Scalar summaries cannot establish that a full profile is reproduced. Functional retention was therefore described for each device by Spearman correlation between $c_{i,\mathrm{aging}}\left(t\right)$ and $c_{i,\mathrm{retest}}\left(t\right)$ across the 56 common bins. In parallel, the device-level conditional stage difference was defined as $\Delta_{i}={\mathrm{median}}_{t}\left\{g_{i,\mathrm{retest}}\left(t\right)-g_{i,\mathrm{aging}}\left(t\right)\right\}$. The former addresses shape after cohort centering; the latter addresses direction and magnitude of a shared-support level difference. Table 2 shows why these quantities answer distinct questions.

### 2.4. Statistical Analysis and Robustness Assessment

Cross-record retention of every scalar feature was summarized by Spearman’s rank correlation ρ. This statistic asks whether devices tend to retain relative order and does not require a linear relation. Numerical agreement was described separately by Lin’s concordance correlation coefficient (CCC), which penalizes departure from both perfect correlation and the identity line [22]. CCC was not interpreted as a formal equivalence test because no metrologically or mechanically justified equivalence margin was available for the uncalibrated output.

Because rank correlation and CCC can diverge for more than one reason, CCC was reported together with the two factors into which it decomposes. Writing *x* and *y* for the paired aging-stage and first-retest values of a feature, with means $m_{x}$ and $m_{y}$ and population standard deviations $s_{x}$ and $s_{y}$,

(4) $$\mathrm{CCC}=r\;C_{b},\;C_{b}=\frac{2}{v+v^{-1}+u^{2}},\;v=\frac{s_{x}}{s_{y}},\;u=\frac{m_{x}-m_{y}}{\sqrt{s_{x}s_{y}}},$$

where *r* denotes the Pearson correlation. The two factors separate the mechanisms that reduce concordance. The precision factor *r* measures how tightly the paired values follow any straight line, whereas the accuracy factor $C_{b}$ measures how far that line departs from the identity line, either through a scale change *v* or through a location shift *u* expressed in units of the geometric mean standard deviation. Reporting *r* and $C_{b}$ alongside CCC therefore identifies which of the two conditions holds when concordance is low: a systematic displacement of the paired values, or a weak association between them. The distinction governs how a low CCC is acted upon in a subsequent test design.

Uncertainty for ρ was quantified with paired, device-level bias-corrected and accelerated (BCa) bootstrap intervals based on 9999 resamples, following the bootstrap framework [32]. Leave-one-device-out correlations were also calculated for the five marginal features to reveal dependence on a single device. Within each defined feature family, two-sided Spearman *p*-values were adjusted with the Benjamini–Hochberg false discovery rate (FDR) procedure [33]. The five marginal features were one family in each cohort; *A*, *W*, and β formed a separate conditioned family. Because asymptotic rank-test values and BCa intervals are not exact duals, interpretation prioritized the device-level interval when they differed; adjusted values were retained as complementary multiplicity-aware descriptors rather than universal pass/fail thresholds. Fixed seed bases of 20260713 and 20261713 generated the primary- and repeat-cohort feature intervals, respectively; seed 41207 generated the conditional-difference interval.

The analysis plan distinguished prespecified structure from findings made within it, and the two are labeled as such throughout. The five marginal features of Section 2.3 were fixed as a single family before the cross-record comparison was run, and all five are reported in full and in the same order for both cohorts; the conditioned family *A*, *W*, and β was fixed in the same way. This prespecification is what makes the family-wise FDR adjustment interpretable and what prevents selective reporting of whichever member proved strongest. The prominence of $Q_{0.05}$ emerged within that prespecified family: no feature was nominated as a primary endpoint in advance, and no feature-specific retention threshold was set before the results were seen. Its status is accordingly provisional. On that basis, the disjoint same-mode cohort was analyzed as an out-of-sample recurrence test, and $Q_{0.05}$ is described below as a candidate endpoint for a controlled retest.

The sign consistency of $\Delta_{i}$ was tested with an exact two-sided binomial sign test under a null positive probability of 0.5. Its median and 95% BCa interval were bootstrapped over devices. Temperature-bin sensitivity was examined at widths of 0.5, 1, and 2 °C. A separate occupancy analysis repeated the 1 °C workflow after requiring at least five and at least ten observations in every retained device–stage–bin. For each threshold, the retained support, direction of $\Delta_{i}$, conditioned scalar correlations, and median complete-profile correlation were recomputed. Inferential transformations and the primary analysis figures were generated with a reproducible workflow using Python 3.12.10, NumPy 2.4.4, pandas 3.0.2, SciPy 1.17.1, and Matplotlib 3.10.8. Companion descriptive graphics were reconstructed from the same processed values and paired CSV records. The processed tables, pseudonymized figure data, and executable workflows are supplied as Supplementary Materials.

## 3. Results

### 3.1. A Retention Signature Rather than a Single Stability Score

Throughout this article, a retention signature denotes the joint pattern that the prespecified features display across the evidence levels of Table 2, read one feature at a time: whether device order is retained in the primary cohort and recurs in the disjoint cohort; whether paired values remain close to the identity line and, where they do not, whether the precision factor *r* or the accuracy factor $C_{b}$ of Equation (4) is responsible; and whether the temperature-conditioned and complete-profile levels support the same reading. The signature is an ordinal description of the evidence each quantity supports. It carries no cut-off on ρ, CCC, *r*, or $C_{b}$: a numerical criterion would require an equivalence margin, and for uncalibrated archived output no metrologically or mechanically justified margin exists. The retention signature is derived in the remainder of Section 3 and summarized after the cross-cohort comparison.

**Figure 2:**
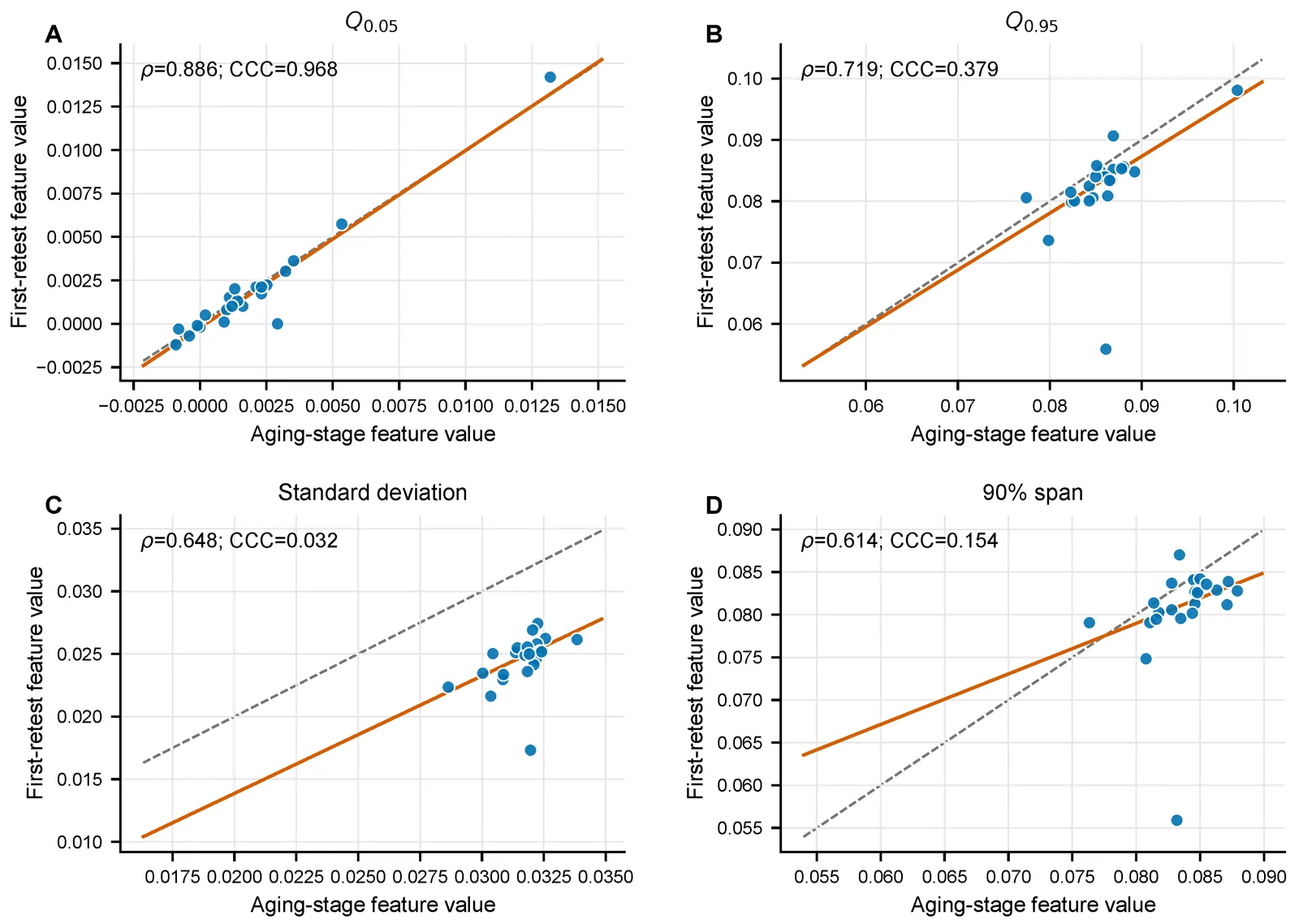
Device-level marginal features in the aging-stage and first-retest records: (**A**) $Q_{0.05}$, (**B**) $Q_{0.95}$, (**C**) standard deviation, and (**D**) the central 90% span. Each point is one paired device. Orange lines are Theil–Sen visual guides; gray dashed lines are identity lines. Spearman ρ and concordance correlation coefficient (CCC) quantify different aspects of retention, and the fitted lines are not calibration models.

The marginal comparisons resolved a feature-specific retention signature. The lower-tail feature $Q_{0.05}$ provided the clearest joint evidence: its device ordering was retained from the aging-stage record to first retest (ρ=0.886, BCa 95% confidence interval (CI) 0.491–0.983; FDR-$q=2.10\times 10^{-7}$), its CCC was 0.968, resolving into a precision factor r=0.973 and an accuracy factor $C_{b}=0.995$, and its median paired change was −0.000201 recorded unit. Thus, the favorable rank result in Figure 2A was accompanied by proximity to the identity line rather than by a common level shift. Leave-one-device-out estimates of ρ remained between 0.869 and 0.970, indicating that this result did not depend on one paired device.

The upper tail and dispersion retained order while showing substantially weaker identity-line concordance, and the decomposition shows that they did so through different mechanisms. For $Q_{0.95}$, ρ was 0.719 (BCa 95% CI 0.351–0.885) whereas CCC was 0.379; because the accuracy factor remained moderate at $C_{b}=0.743$, the reduction is attributable mainly to precision (r=0.510), with the paired values remaining near the identity line. Standard deviation exhibits the opposite mechanism: ρ=0.648 (0.328–0.838) but CCC = 0.032, with r=0.420 and $C_{b}=0.077$. Its near-zero concordance is therefore almost entirely an accuracy failure, driven by a location shift of u=4.84 geometric mean standard deviations, and the point cloud is displaced from the identity line accordingly (Figure 2C); its median paired change was −0.006887 recorded units. $D_{90}$ follows the $Q_{0.95}$ mechanism but is weaker on both factors (ρ=0.614, CCC = 0.154, r=0.264, $C_{b}=0.583$). The corresponding leave-one-device-out ranges were 0.677–0.803, 0.612–0.717, and 0.568–0.679, respectively. A precision-limited feature and a displacement-limited feature both present as a low CCC, yet they call for different controls in a confirmatory test, as Section 4.3 sets out.

The unconditioned median supplied a third pattern. Its rank estimate was smaller and uncertain (ρ=0.380, BCa 95% CI −0.077–0.700; FDR-q=0.0808), and its CCC was 0.048, with r=0.682 and $C_{b}=0.071$ at a location shift of u=5.12. Its precision factor was thus the second largest of the five features while its rank correlation was the smallest, a combination consistent with a limited number of high-leverage devices sustaining the linear association without a correspondingly stable ordering. The low concordance again reflects systematic displacement, with covariation preserved. Central location was therefore not supported as a transferable device attribute under the two archived procedures. Taken together, the five features show why neither rank nor concordance alone can represent cross-record stability. Table 3 places these primary-cohort contrasts alongside the disjoint repeat-cohort results.

**Figure 3:**
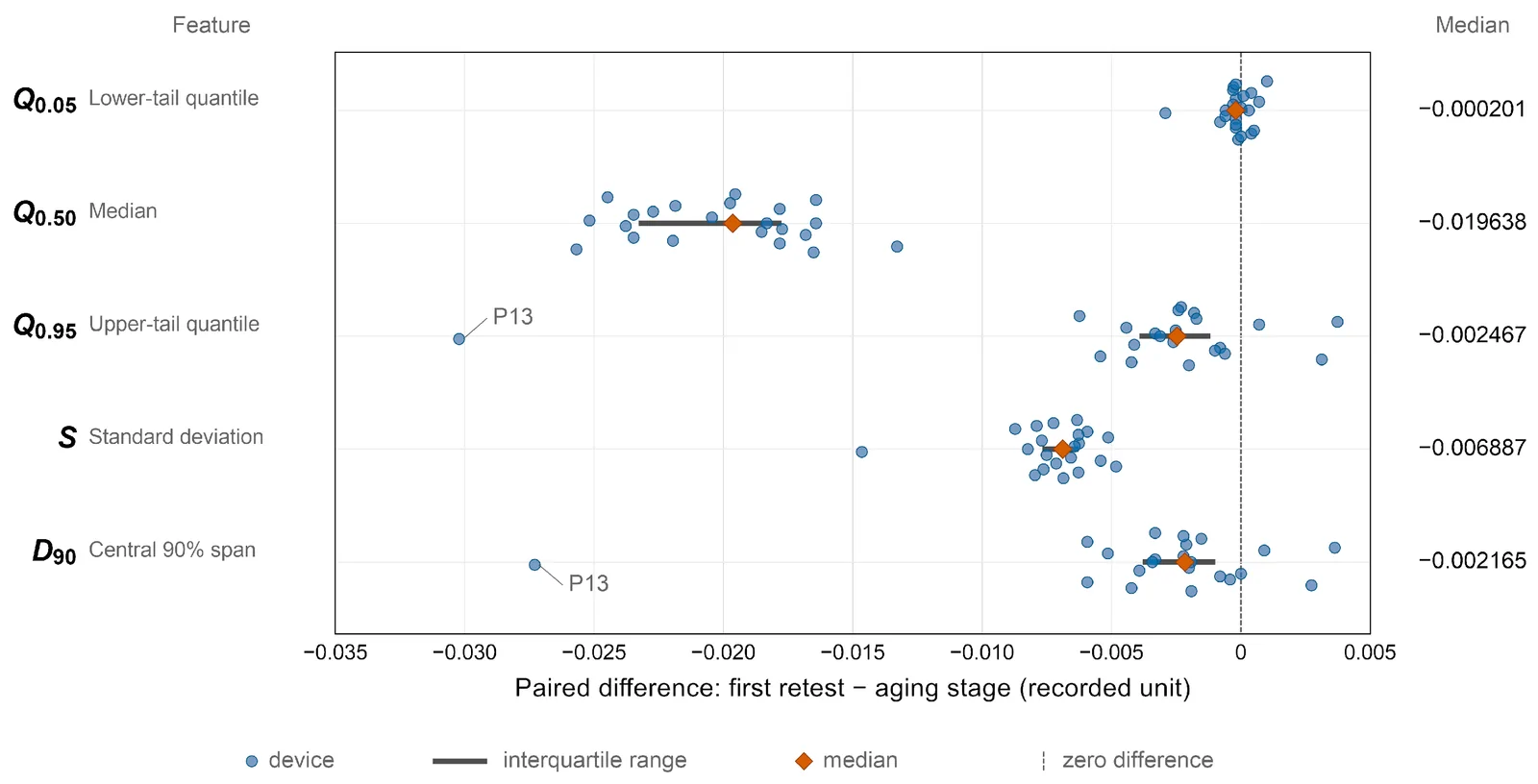
Shared-scale distributions of first-retest-minus-aging differences for five marginal features in the primary cohort (n=22 devices). Blue circles represent devices, dark horizontal segments show interquartile ranges, orange diamonds show medians, and the gray dashed line denotes zero difference. All features are displayed on the same recorded-unit axis. P13 is labeled because the same device produced the marked negative $Q_{0.95}$ and $D_{90}$ differences. The plot describes the magnitude and pattern of disagreement; it does not define limits of agreement or equivalence bounds.

**Figure 4:**
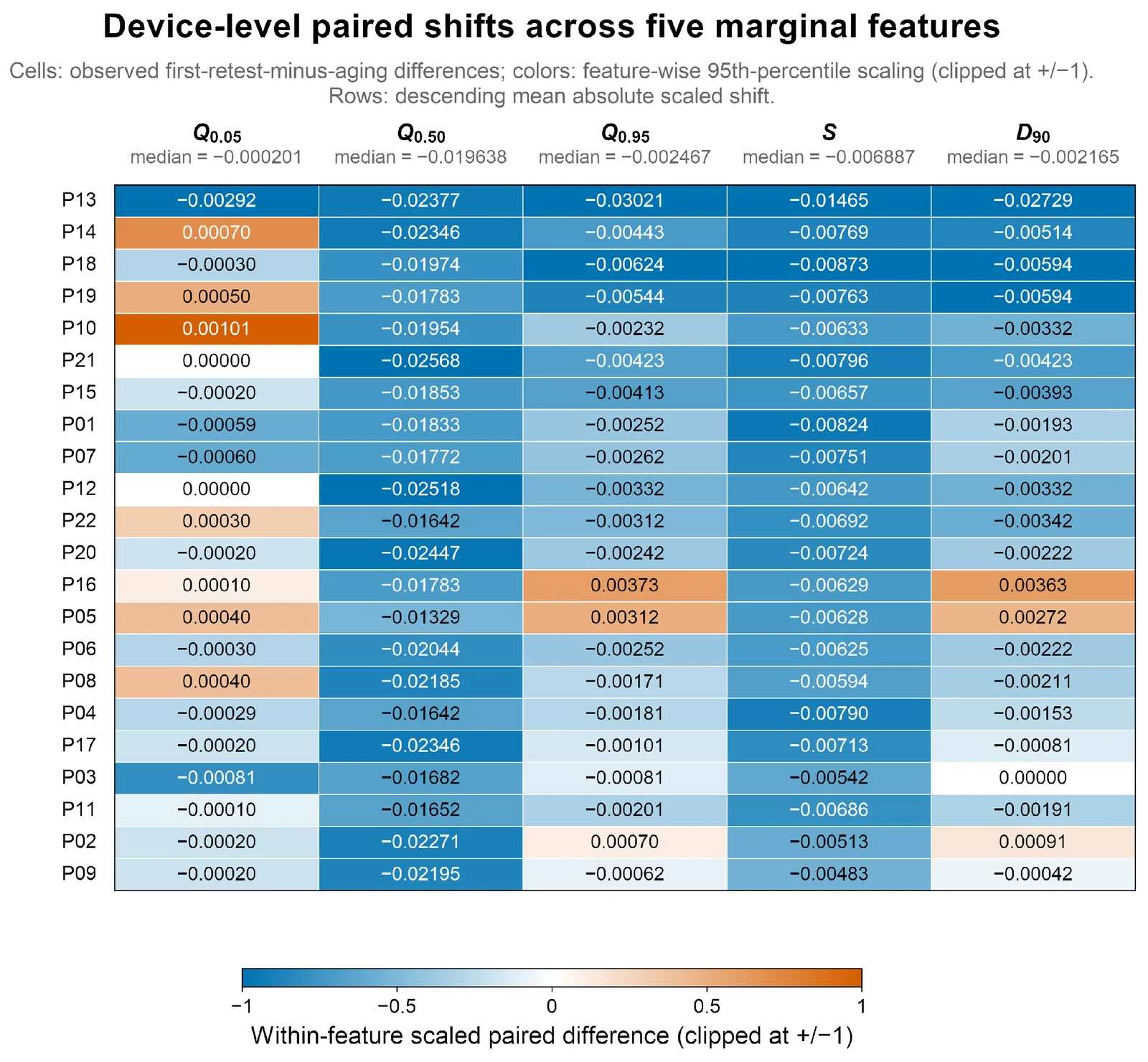
Device-level paired changes in five marginal features for the primary cohort. Cells report first-retest-minus-aging differences in recorded units; column headings report feature medians. Colors are scaled separately within each feature by its 95th percentile absolute difference and clipped at ±1, and rows are ordered by decreasing mean absolute scaled shift. The color intensity is therefore comparable within, but not numerically across, feature columns.

The shared-scale paired-difference distributions clarify how identity-line disagreement arose (Figure 3). $Q_{0.05}$ differences were concentrated near zero, with a median of −0.000201 recorded unit and values on both sides of zero. By contrast, all 22 devices had negative $Q_{0.50}$ and *S* differences, indicating cohort-wide displacement rather than an effect confined to a narrow range of stage means. Most $Q_{0.95}$ and $D_{90}$ differences remained much closer to zero, but the same pseudonymized device (P13) had markedly negative differences for both features. The device–feature matrix in Figure 4 makes these two forms explicit and shows why positive rank retention can coexist with either a broadly shared displacement or a device-specific upper-tail departure. The distributions are descriptive agreement diagnostics; formal limits of agreement were not estimated because the two archived procedures were not replicate measurements under one controlled protocol.

### 3.2. Temperature Support Reverses the Apparent Record Difference

**Figure 5:**
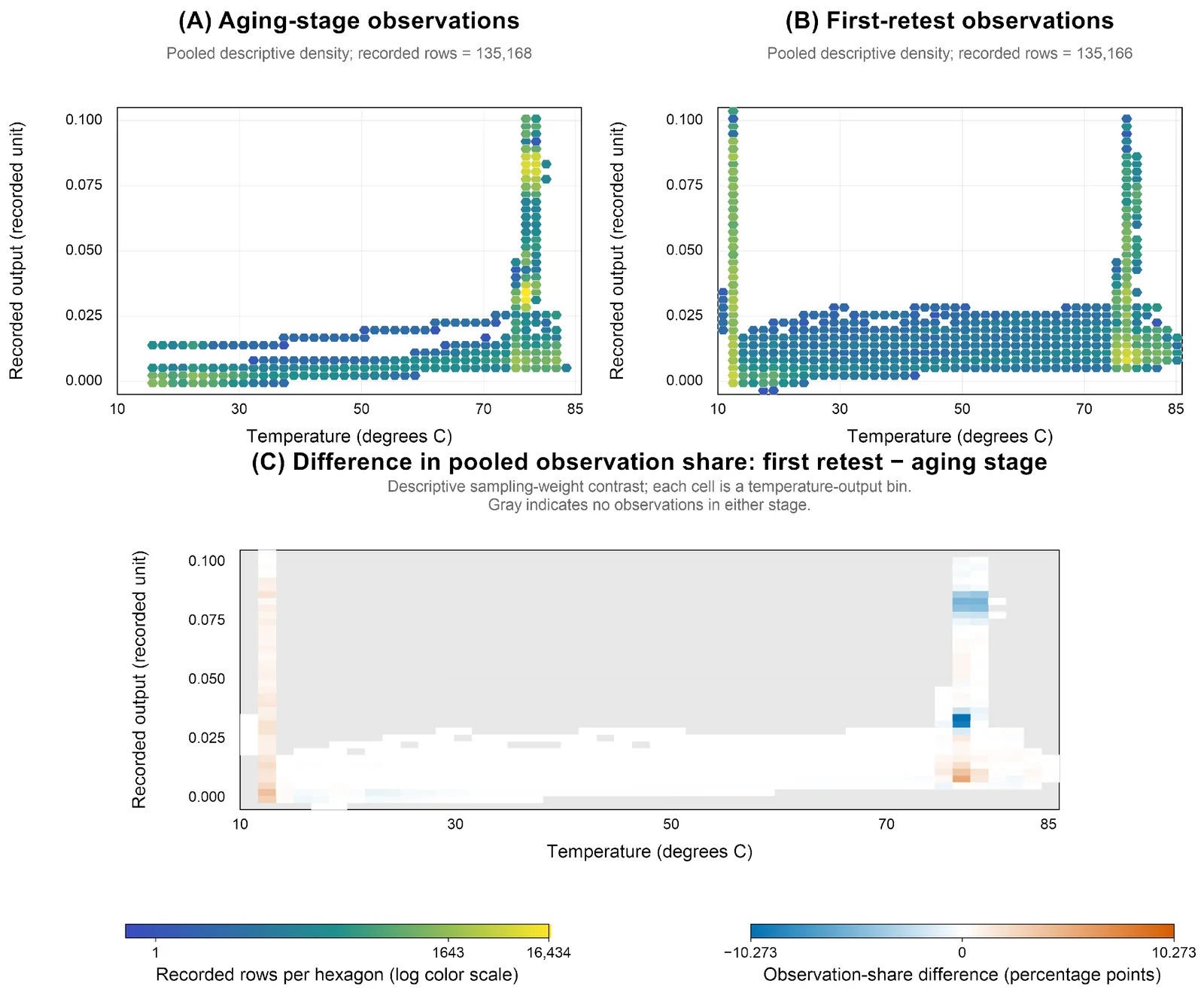
Pooled descriptive temperature–output densities for the primary cohort. (**A**) Aging-stage observations (135,168 recorded rows); (**B**) first-retest observations (135,166 recorded rows); and (**C**) first-retest-minus-aging difference in pooled observation share for each temperature–output cell. Panels (**A**,**B**) use a common logarithmic count scale; gray cells in panel (**C**) contain no observations at either stage. Recorded rows describe empirical sampling density and were not treated as independent inferential replicates.

The sign of the stage comparison depended on how temperature support entered the estimand. The pooled temperature–output densities reveal markedly different empirical sampling weights, particularly near the low-temperature boundary and within the upper-temperature cluster (Figure 5); the unconditioned summaries therefore describe stage-specific observation weighting rather than a matched-temperature contrast. Using the original observation weighting, every device had a negative first-retest-minus-aging median difference, and the median device-level difference was −0.019638 recorded units. Restricting both records to 56 common temperature bins and assigning equal weight to each retained bin reversed the sign for all 22 devices: the median conditional difference was 0.003978 recorded units (BCa 95% CI 0.003298–0.008887), the range was 0.001108–0.018505, and the exact sign-test result was $p=4.77\times 10^{-7}$. This dependence is examined below using a device–temperature map and paired raw-to-conditioned comparisons. The inferred record difference therefore depends jointly on the retained temperature domain and empirical temperature weighting; the remaining positive difference cannot be identified as a calibration offset or aging effect.

The conditioned curves also localized a boundary-dependent discrepancy. Both cohort profiles rose gradually over most of the common domain, but the aging-stage median had a sharp excursion near the upper limit that was much smaller at retest. The across-device median difference was negative at 76–79 °C and returned to a positive value at 80 °C. This excursion is treated as a procedure-boundary feature; the occupancy evidence supporting that reading, and the specific sense in which the term is used, are set out after the device–temperature map below.

**Figure 6:**
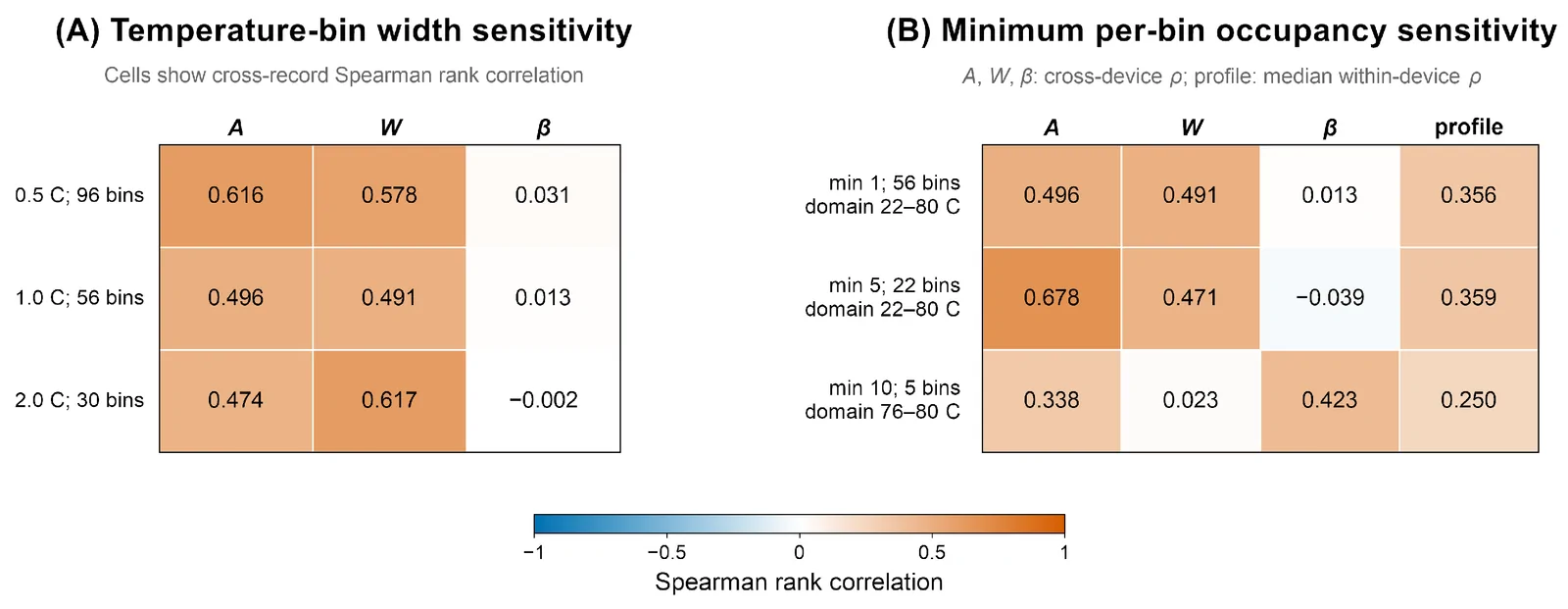
Sensitivity of temperature-conditioned retention to analytical support choices. (**A**) Cross-record Spearman rank correlations for relative offset *A*, centered span *W*, and relative temperature slope β at bin widths of 0.5, 1, and 2 °C; row labels give the number of retained bins. (**B**) Corresponding correlations under minimum per-bin occupancies of 1, 5, and 10 observations; the profile column reports the median within-device complete-profile correlation, and row labels give the retained bin count and temperature domain. Both panels use the same −1 to 1 correlation scale. The five-bin, ten-observation result describes only the 76–80 °C boundary band and is not a whole-domain retention estimate.

Centering each device against its leave-one-out cohort reference separated persistent relative level from temperature-dependent shape. Relative offset *A* had ρ=0.496, although its BCa interval included zero (−0.028–0.773; FDR-q=0.0306). The centered span *W* had ρ=0.491 (0.005–0.794; FDR-q=0.0306). Relative temperature slope was not retained (ρ=0.013, −0.526–0.558; FDR-q=0.954). Varying bin width from 0.5 to 2 °C preserved this ordering (Figure 6A): ρ ranged from 0.474 to 0.616 for *A*, from 0.491 to 0.617 for *W*, and from −0.002 to 0.031 for β.

The occupancy analysis distinguished sensitivity to sample support from sensitivity to bin width (Figure 6B). Requiring at least five observations from every device and stage retained 22 common bins spanning 22–80 °C. In total, 21 of 22 device differences remained positive, with a median of 0.004028 recorded units. Estimates for *A*, *W*, β, and the median complete-profile correlation were 0.678, 0.471, −0.039, and 0.359, respectively, broadly preserving the primary pattern. Requiring ten observations left only five bins, all at 76–80 °C. All device differences then became negative, and the median was −0.019789 recorded units. The ten-observation rule changed the estimand rather than merely tightening it, because the retained domain collapsed to the upper-boundary region. Because only five upper-boundary bins remained, the associated scalar and profile correlations are reported descriptively in the Supplementary Materials but are not interpreted as whole-domain retention. A conditional stage difference must therefore always be reported with its retained temperature domain.

**Figure 7:**
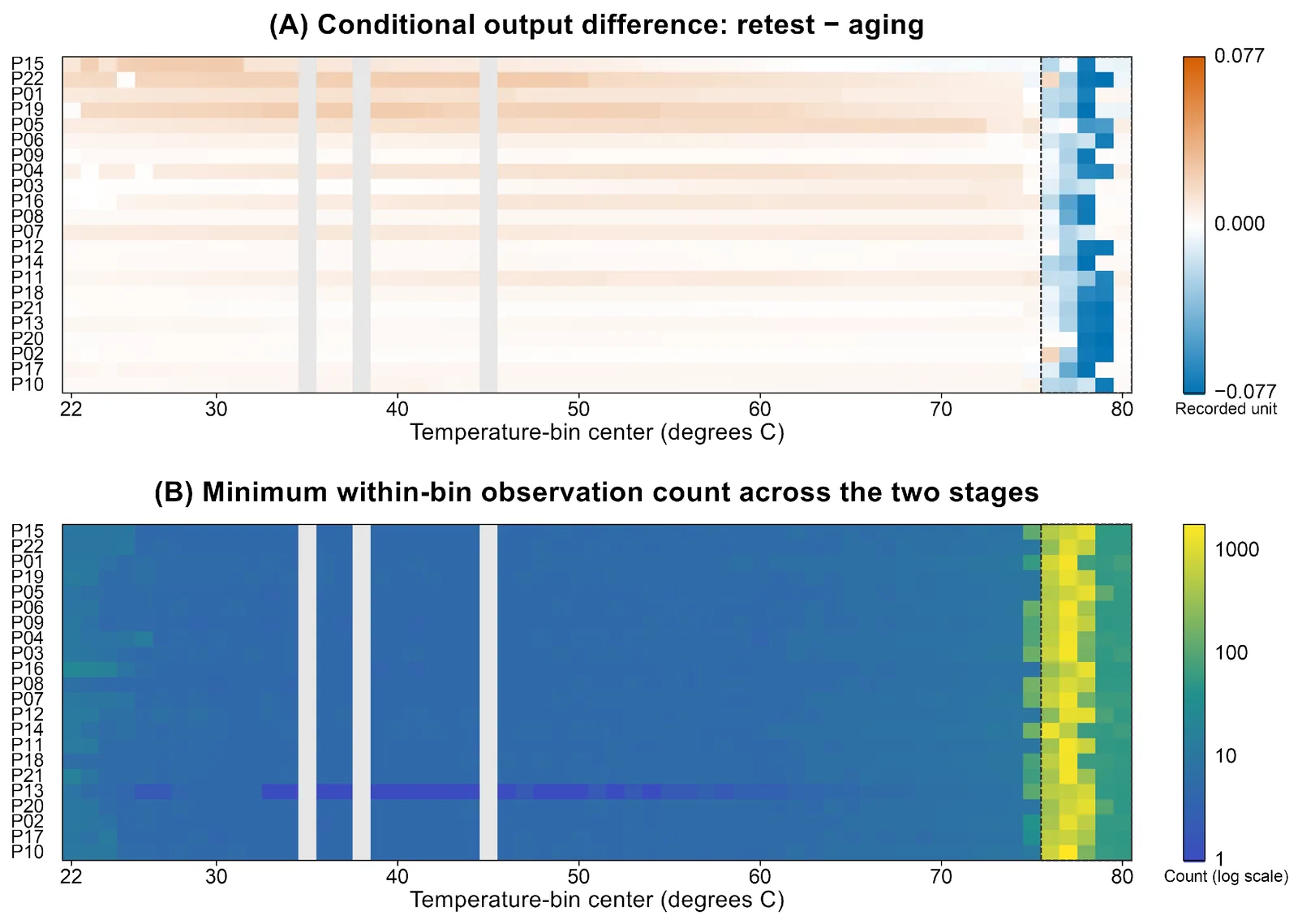
Device–temperature structure on the one-degree common-support grid for the primary cohort. (**A**) Conditional output difference $g_{i,\mathrm{retest}}\left(t\right)-g_{i,\mathrm{aging}}\left(t\right)$ in recorded units, shown on a zero-centered symmetric color scale spanning the largest observed absolute difference. (**B**) Minimum observation count across the two stages for each device–bin, shown on a logarithmic color scale. Rows are pseudonymized devices ordered from the lowest to the highest complete-profile correlation. Gray columns at 35, 38, and 45 °C lack complete common support; dashed boxes mark the 76–80 °C band retained by the ten-observation rule.

The device–temperature map locates this dependence on support (Figure 7). Across the common cells at 22–75 °C, 98.7% of conditional differences were positive, and 93.2% had a minimum two-stage occupancy below ten observations. Counts rose sharply at 76–80 °C, where 78 of 110 device–bin differences were negative. The ten-observation rule therefore selected a high-occupancy boundary band rather than a denser representation of the full common domain. The absent columns at 35, 38, and 45 °C further show that complete support was discontinuous and was not filled by interpolation.

**Figure 8:**
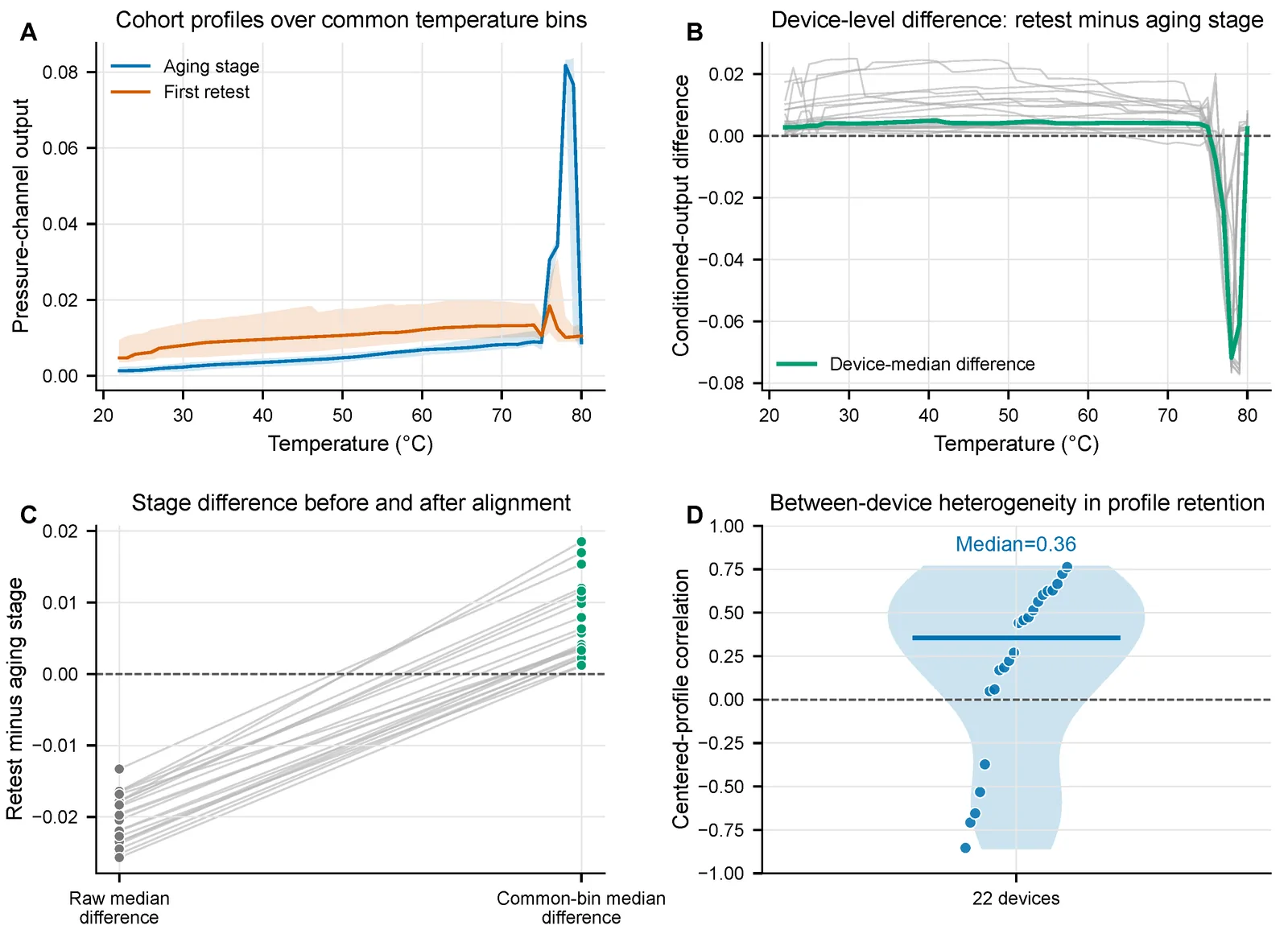
Temperature-conditioned comparison on 56 bins shared by every primary-cohort device and both stages. (**A**) Cohort median profiles with interquartile bands. (**B**) Individual retest-minus-aging differences (gray) and their device median (green). (**C**) Device-level stage differences before and after common-bin alignment. (**D**) Distribution of within-device correlations between complete centered profiles. The excursion at 76–79 °C is retained as an observed procedure-boundary feature, that is, an excursion localized at the upper edge of the temperature range the archived procedure visited and coinciding with a sharp rise in observation counts there; it is not interpreted as an intrinsic device response.

The term procedure-boundary feature denotes an excursion localized at the edge of the temperature range the archived procedure visited, whose magnitude tracks how that edge was sampled. Three observations support that reading for the 76–79 °C excursion (Figure 8A,B). First, the band lies immediately below the upper extent of both records, 82.8 °C in the aging-labeled stage and 85.1 °C at first retest, so it marks the approach to the highest temperature reached, at the edge of the domain. Second, occupancy changes character there: across 22–75 °C, 93.2% of device–bins hold fewer than ten two-stage observations, whereas counts rise sharply at 76–80 °C. A concentration of observations confined to the top of the range is characteristic of a hold at a set point. Third, the sign of the conditional difference inverts within that band and only there, 78 of 110 device–bin differences being negative against 98.7% positive over 22–75 °C. A device-intrinsic change would not be expected to reverse precisely where the procedure dwelt.

These observations are consistent with the two record groups having approached and held the upper set point differently, whether in ramp rate, dwell duration, or degree of thermal equilibration. A high-temperature response change, a self-heating or thermal-lag transient, and a difference in chamber control would each produce a similar signature, and separating them calls for the set-point program, dwell times, and an explicit equilibrium criterion listed in Section 4.4. The excursion is therefore reported as an observed property of the archived procedures at their boundary and is kept outside device-level interpretation.

Complete centered profiles were more heterogeneous than any scalar summary. Their median within-device correlation was 0.356, with an interquartile range of 0.051–0.593 and a range from −0.854 to 0.763; in total, 17 of 22 correlations were positive. A device could therefore preserve a lower-tail or dispersion rank without retracing its cohort-relative response over temperature. The scalar and functional findings answer different questions and cannot be combined into one stability label.

### 3.3. Cross-Cohort Recurrence and the Boundary of Transfer

The disjoint same-mode cohort served as a corroborating stress test of marginal-feature ordering. Positive rank retention recurred for $Q_{0.05}$ (ρ=0.624, BCa 95% CI 0.231–0.860; FDR-q=0.0164), $Q_{0.95}$ (ρ=0.756, 0.421–0.925; q=0.00353), and standard deviation (ρ=0.691, 0.130–0.910; q=0.00756). These were three of the four marginal features with supported rank retention in the primary cohort; $D_{90}$, despite positive primary-cohort evidence, did not recur in the disjoint cohort. The wider intervals are consistent with the smaller cohort, while the spacing between retained records remained uncontrolled.

**Figure 9:**
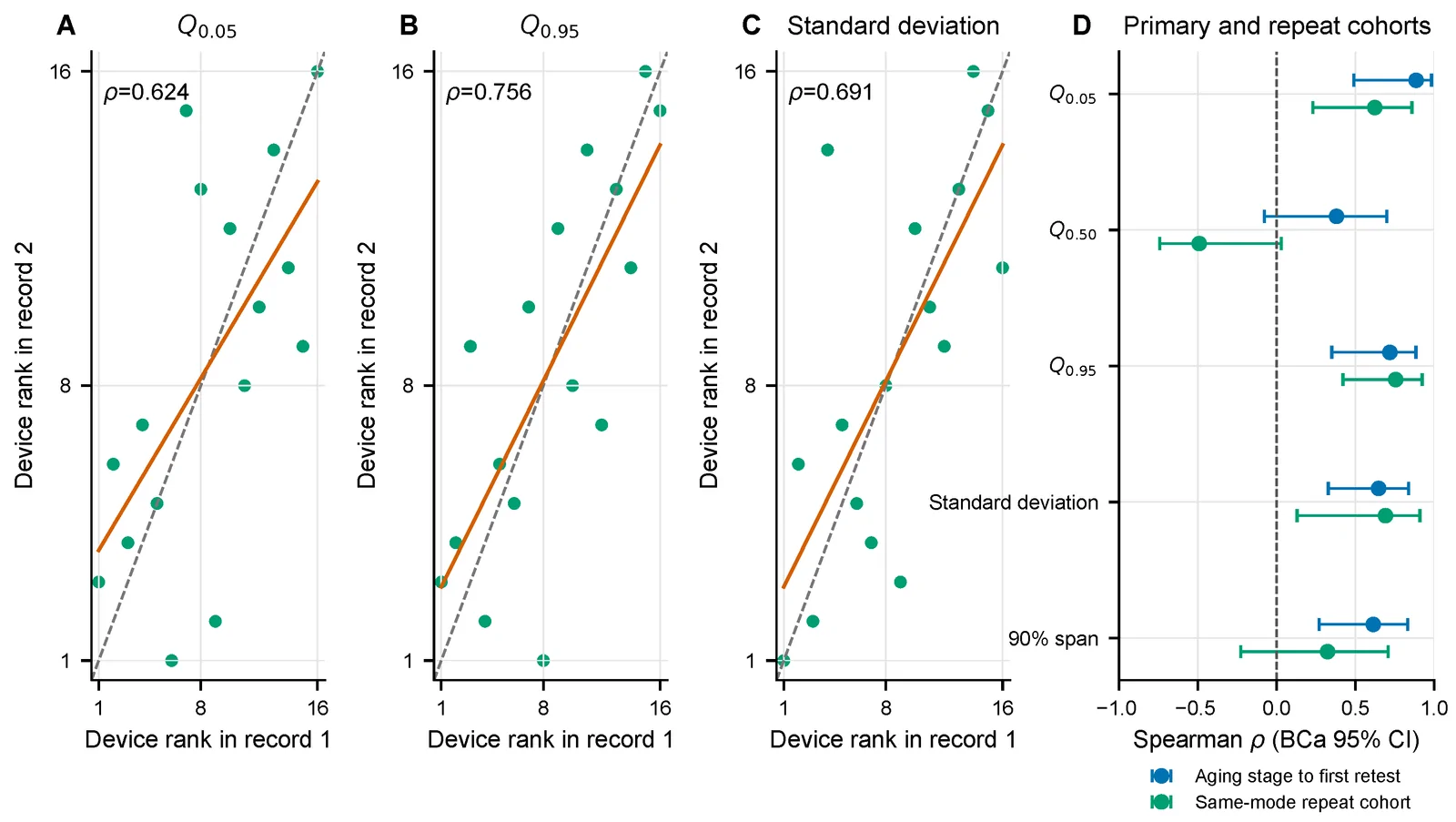
Cross-cohort recurrence of marginal-feature ordering. Panels (**A**–**C**) show device ranks for $Q_{0.05}$, $Q_{0.95}$, and standard deviation in the two same-mode records; orange lines are Theil–Sen visual guides and dashed lines are identity lines. Panel (**D**) compares Spearman estimates and bias-corrected and accelerated (BCa) 95% intervals between the primary and disjoint repeat cohorts.

That recurrence did not extend to all marginal features. $Q_{0.50}$ had a negative point estimate (ρ=−0.491, BCa 95% CI −0.741–0.030; q=0.0667), and $D_{90}$ had ρ=0.324 with an interval crossing zero (q=0.222). Figure 9D therefore separates a cross-cohort hierarchy—positive lower-tail, upper-tail, and dispersion ordering—from cohort-dependent median and combined-span results. Thus, the transferable object is the relative rank hierarchy, not numerical level, temperature-conditioned behavior, or aging-related evidence.

**Figure 10:**
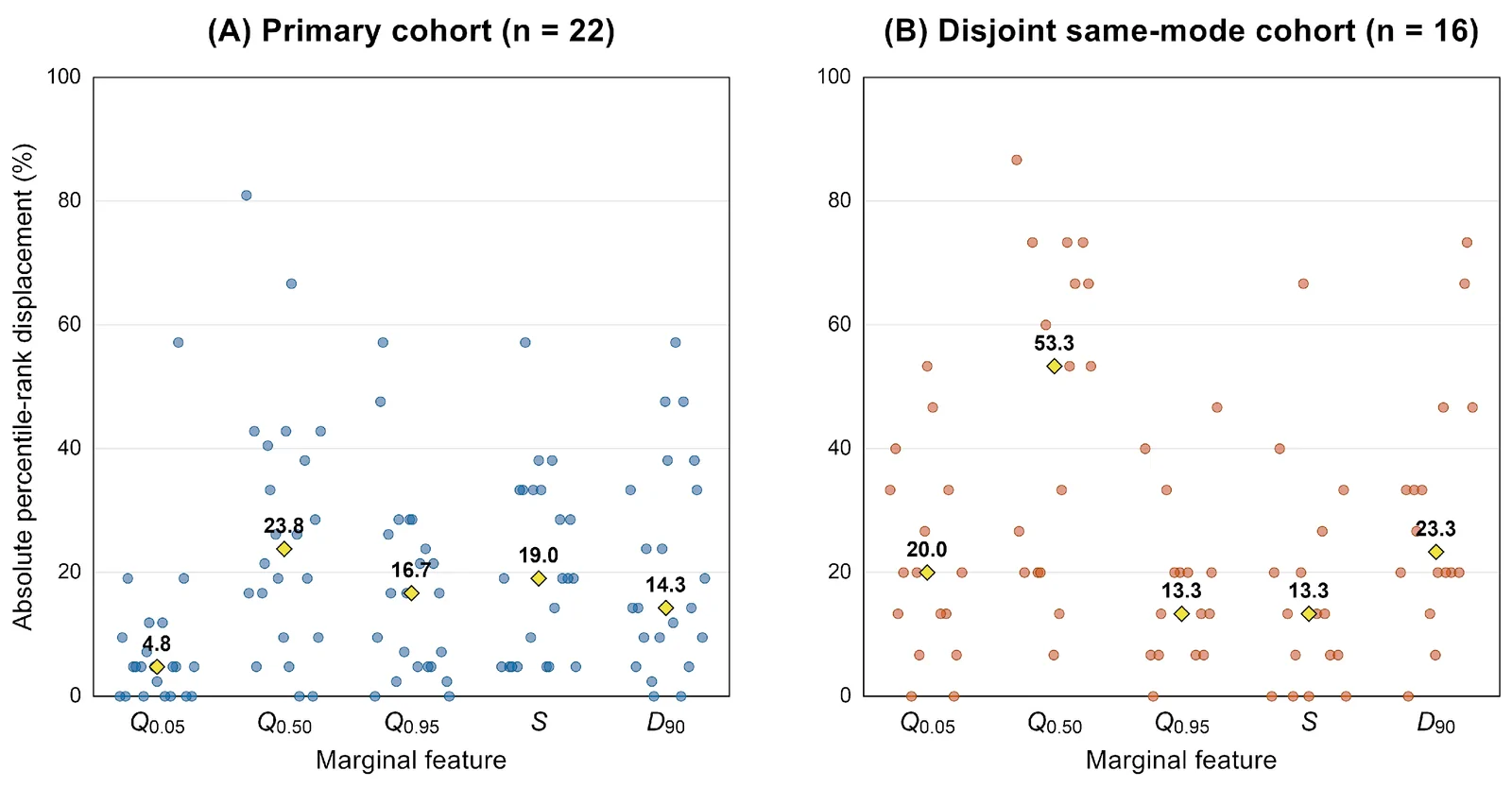
Device-level absolute percentile-rank displacement for five marginal features in (**A**) the primary cohort (n=22) and (**B**) the disjoint same-mode cohort (n=16). Displacement was calculated as $100|r_{2}-r_{1}|/\left(n-1\right)$ using average ranks for ties. Dots represent devices; yellow diamonds and adjacent values show feature medians. The distribution reveals device-level rank movement that is not visible from a single cohort correlation.

Rank displacement provides a device-level complement to the cohort correlations (Figure 10). In the primary cohort, median absolute movement was lowest for $Q_{0.05}$ (4.8%) and higher for $Q_{0.50}$ (23.8%). In the disjoint cohort, $Q_{0.95}$ and *S* each had a median movement of 13.3%, whereas the $Q_{0.50}$ median rose to 53.3%. This contrast reinforces the recurrent upper-tail and dispersion ordering while exposing the loss of median ordering at the device level. Individual devices nevertheless moved much farther than the median in every feature. Because absolute displacement omits direction and the joint rank pattern, it complements rather than replaces Spearman ρ; the primary $D_{90}$ median of 14.3%, for example, did not guarantee recurrence in the second cohort.

**Figure 11:**
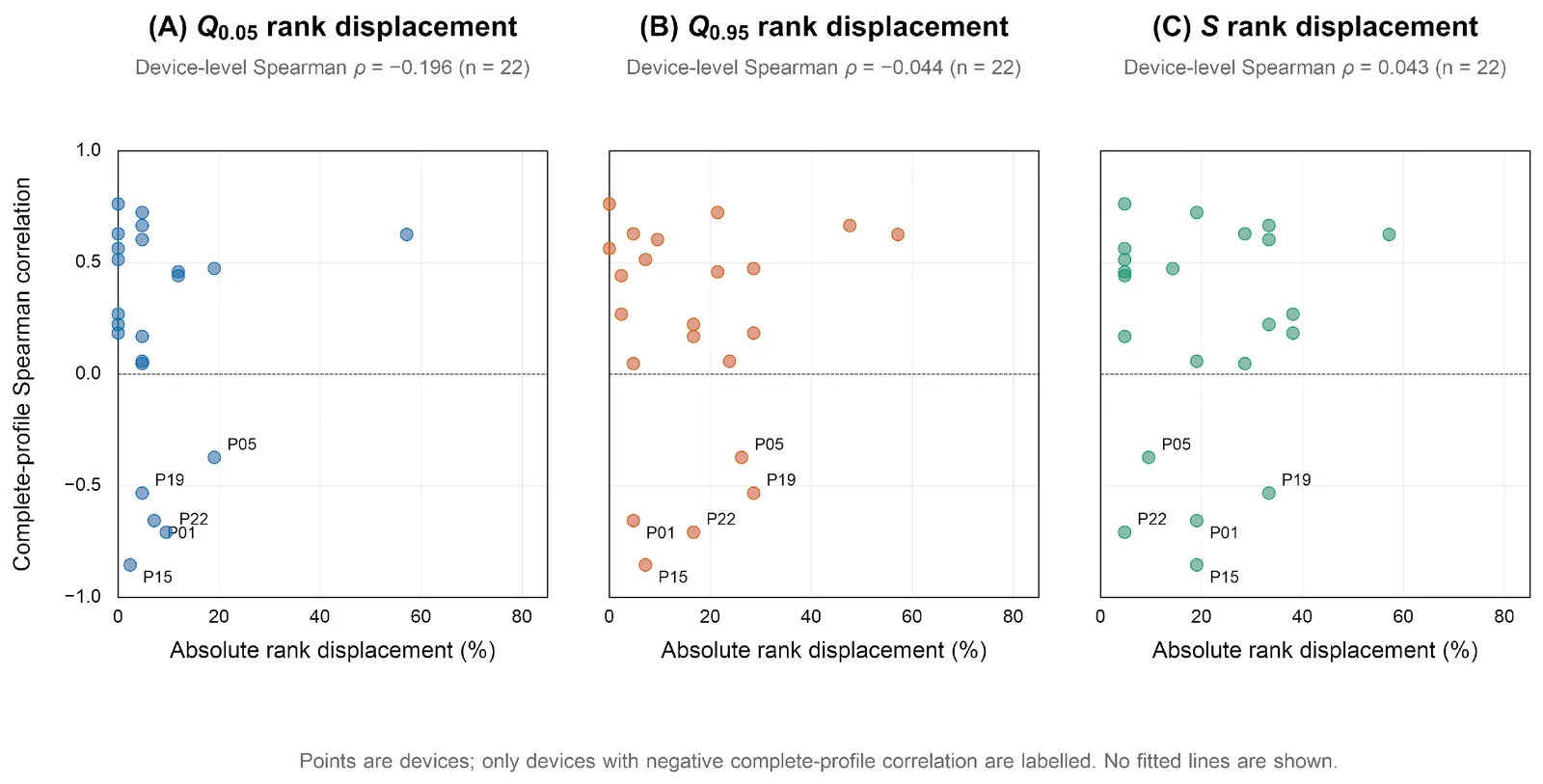
Relationship between scalar rank movement and functional profile retention in the primary cohort (n=22 devices). Panels (**A**–**C**) compare absolute percentile-rank displacement for $Q_{0.05}$, $Q_{0.95}$, and *S*, respectively, with the within-device Spearman correlation between complete centered profiles. Panel annotations give the device-level Spearman correlation between the two axes; devices with negative profile correlation are labeled, and the dashed line denotes zero profile correlation. No fitted relation or causal interpretation is imposed.

Within the primary cohort, absolute rank displacement showed little monotonic relation to complete-profile retention for $Q_{0.05}$, $Q_{0.95}$, or *S* (Spearman ρ=−0.196, −0.044, and 0.043, respectively; Figure 11). Negative profile correlations also occurred among devices with modest scalar rank movement. Scalar ordering and functional reproduction therefore remain distinct evidence objects.

Table 4 collects these results into the signature defined at the start of Section 3. Read across rows, it shows that no feature is retained at every level and that the levels disagree in specific and interpretable ways: $Q_{0.05}$ is the only feature supporting both order and numerical agreement; $Q_{0.95}$ and *S* support order alone but lose concordance through different factors—precision and accuracy, respectively; $D_{90}$ retains order within the primary cohort but does not recur; and $Q_{0.50}$ supports neither. Panel (B) adds that the temperature-conditioned and functional levels do not reproduce the marginal result: the relative temperature slope is not retained, complete profiles remain heterogeneous, and the sign of the conditional stage difference is a property of the retained temperature domain. The signature is the entire pattern, and each cell of this table carries its meaning from the row it sits in.

## 4. Discussion

### 4.1. Metrological Meaning of the Retention Signature

The principal result is not a division between stable and unstable features; it is a separation of metrological questions that a single coefficient conflates. Rank correlation assesses whether the device order persists, CCC quantifies the extent to which paired values remain close to the identity line, and functional correlation describes whether a cohort–relative curve retraces its shape. Their divergence is therefore informative rather than contradictory.

Decomposing CCC sharpens that reading, because a low value by itself carries no single meaning. Standard deviation and $Q_{0.95}$ both retained device order and both lost concordance, yet they did so for opposite reasons. For *S*, the accuracy factor collapses ($C_{b}=0.077$) under a location shift of nearly five geometric mean standard deviations while the association remains moderate (r=0.420): a device that was relatively variable tended to remain relatively variable, but the amount of variability moved cohort-wide. For $Q_{0.95}$, the accuracy factor is largely intact ($C_{b}=0.743$), and the binding limitation is precision (r=0.510); paired values are not systematically displaced; they are scattered. The consequence for a subsequent test differs accordingly. A displacement-limited feature may remain usable once a traceable reference fixes the level, because a cohort-wide component is a candidate for correction; a precision-limited feature reflects device-to-device irreproducibility, and it would require replicated readings at fixed reference states before serving as an endpoint. The two therefore separate at the point where a test protocol is chosen.

The lower tail is the only feature that combined high primary-cohort concordance with positive ordering in both cohorts. This makes $Q_{0.05}$ a plausible endpoint for a controlled follow-up, but not yet an intrinsic fingerprint, and two conditions bound that status. Its prominence emerged within the prespecified feature family (Section 2.4), so the disjoint-cohort recurrence corroborates a finding, and a prospective study would nominate $Q_{0.05}$ before the data are seen. Its value may depend on minimum applied load, an acquisition floor, loading direction, and residence time in the low-output part of the path, each of which a controlled protocol would fix. The upper tail and standard deviation provide weaker evidence of persistent device structure because their ranks recurred while their primary-cohort levels moved. The negative or uncertain median result further indicates that the center of an observation-weighted record is especially sensitive to the procedure used to generate it.

This interpretation changes how archived sensor comparisons should be read. A retained order may be useful for prioritizing candidate endpoints or detecting whether a batch hierarchy persists; it cannot by itself establish interchangeability, accuracy, or a permissible change. Conversely, poor concordance need not imply that all device-specific information has been lost. The multilevel framework preserves this distinction and makes the next measurement requirement visible. The contrast with the controlled long-term evidence surveyed in the Introduction is instructive: there, a defined pressure and temperature protocol supports a quantitative stability figure, whereas the archived records here support a statement about retained structure and nothing beyond it.

### 4.2. Temperature Support, Boundary Behavior, and Response Shape

The raw-to-conditioned sign reversal is a support problem with direct sensor-testing consequences. An unconditioned median weights the temperatures, pressure states, and dwell durations actually visited in a record. Restricting the comparison to a common domain and weighting its temperature bins equally changed both support and thermal weighting, and reversed every paired comparison. The result establishes that the original marginal shift cannot be interpreted independently of thermal coverage. It does not show that temperature alone caused the difference, because pressure input, path direction, and time remain unaligned.

The occupancy sensitivity sharpens that conclusion. A five-observation rule reduced the number of common bins but preserved coverage across the full 22–80 °C span and largely preserved both the sign and the functional summaries. The ten-observation rule retained only the 76–80 °C boundary and produced the opposite sign. This is not evidence that one count threshold is universally correct. It shows that a seemingly stricter support rule can silently redefine the temperature domain, allowing a local procedure-dependent excursion to dominate the estimand. Reporting the bin rule without the retained domain would therefore be insufficient.

The weak relative-slope retention and wide distribution of complete-profile correlations further show that temperature alignment does not make the two records functionally interchangeable. High-temperature pressure-sensor characterization defines thermal hysteresis through different outputs at the same temperature on heating and cooling paths and quantifies it under an explicit thermal cycle [34]. Packaging-generated hygro-thermo-mechanical stress can also affect high-precision MEMS pressure output and temperature hysteresis [35], so stage-dependent shape has physically credible sources. The archived evidence cannot distinguish such effects from chip response, assembly, pressure path, dwell, or chamber procedure. The data identify where cross-record agreement weakens but do not resolve the responsible physical pathway.

### 4.3. From Archived Comparison to a Controlled Retest Design

The findings imply a staged retest rather than one omnibus stability statistic. Candidate marginal endpoints should first be examined for order retention and then for identity-line agreement against a prospectively defined tolerance. Static and dynamic pressure calibrations address different response properties, so the loading waveform and bandwidth must be declared rather than treated as interchangeable test details [36]. Temperature-conditioned differences should be calculated only on a declared domain with controlled coverage and adequate occupancy. Complete profiles should then test whether retained scalar features conceal path-dependent changes. This sequence connects the statistical contrast to a concrete measurement decision at each level.

Together, the recurrent ranks, the two distinct routes to weak CCC, the sign reversal, and the profile heterogeneity specify five distinct retest controls (Table 5). The repeated ordering of $Q_{0.05}$, $Q_{0.95}$, and *S* identifies features worth taking forward, while poor CCC specifies the need for traceable reference pressure and an engineering equivalence margin. Which control is binding depends on which factor of Equation (4) is limiting: displacement-limited features such as *S* and $Q_{0.50}$ require a traceable level reference against which a cohort-wide offset could be estimated and, if warranted, corrected, whereas precision-limited features such as $Q_{0.95}$ and $D_{90}$ require replication at fixed reference states, because their disagreement is device-specific scatter that no offset removes. The sign reversal specifies control of temperature path, dwell, and support. Profile heterogeneity specifies repeated ascending and descending paths with an explicit equilibrium criterion. These controls form a prospective design bridge rather than a retrospective acceptance rule.

In a routine workflow, the same hierarchy could be used as a diagnostic screen before calibration: rank for batch structure, concordance and paired change for level agreement, support alignment for procedural comparability, and profile analysis for path dependence. Device rejection thresholds, however, would require prospective reference data, repeat operators, and a population chosen for that decision. The present analysis defines what must be measured before such thresholds can be justified.

### 4.4. Limitations, Evidence Boundary, and Confirmatory Requirements

Three classes of conclusion sit outside the scope set in Section 2.2, and each is reached by adding a specific measurement. Sensor life and remaining useful life follow from a time base: the storage or operating duration preceding the aging-stage record and the elapsed interval before first retest (Table 1), which together yield a rate of change per unit time. Degradation of sensitivity follows from synchronized reference pressure at both stages, since sensitivity is the response of output to applied pressure. Retention of metrological characteristics follows from traceable reference values together with a controlled and documented loading path, which give accuracy, nonlinearity, hysteresis, and repeatability. The features analyzed here are descriptors of archived output distributions, and a retained rank or a high CCC is evidence of cross-record structural persistence.

The unconfirmed output scale, absent reference pressure, and undocumented exposure therefore restrict inference to cross-record structure. Self-calibrating MEMS architectures illustrate one route to an internal reference by generating a known cavity-pressure stimulus and comparing it with sensor output [37]. Missing timestamps and loading sequence likewise preclude separation of short-term repeatability, elapsed-time drift, path effects, and sensor response.

The number of independent units is also modest: 22 primary devices and 16 disjoint repeat devices. Dense rows improve within-record feature estimation but do not increase these sample sizes. Moderate associations consequently have wide device-level intervals. Complete-bin conditioning avoids extrapolation, but it removes unsupported temperatures and does not equalize within-bin pressure, dwell, or observation count. The occupancy analysis reveals rather than removes this dependence on domain. The repeat cohort adds evidence about the recurrence of rank hierarchy only; its different scale, devices, and file intervals prevent numerical pooling or aging inference.

A confirmatory program should synchronize sensor output with traceable reference pressure and time, record chamber and chip temperature where available, and preserve loading direction, dwell, operating mode, package or lot, and exposure history. Repeated pressure levels and thermal ramps would permit estimation of offset, sensitivity, nonlinearity, hysteresis, repeatability, and drift. A prospectively defined aging exposure together with an unexposed or reference cohort would then enable causal contrasts. High-temperature and high-pressure downhole-tool test systems already provide a basis for controlled environmental loading and synchronized acquisition [38]; repeated device-level calibration would connect the retention signature developed here to defensible accuracy and reliability endpoints.

## 5. Conclusions

The archived comparisons yielded a multilevel retention signature rather than a general stability property. In the primary cohort, $Q_{0.05}$ combined strong rank retention with a high CCC point estimate, and its positive ordering recurred in a disjoint same-mode cohort. $Q_{0.95}$ and standard deviation also retained order in both cohorts, although their lower primary-cohort CCC estimates indicated weaker identity-line agreement; decomposing CCC showed that the two reached that outcome by different routes, $Q_{0.95}$ through limited precision (r=0.510) and standard deviation through a systematic level displacement ($C_{b}=0.077$). Median output and $D_{90}$ did not recur consistently. These contrasts demonstrate that device ordering, numerical agreement, and response shape are distinct evidence objects, and that a single concordance value does not identify which of them has failed.

The retained temperature domain materially changed the inferred record difference. Restricting both records to 56 shared bins and weighting those bins equally reversed every device’s raw-median comparison. Under the five-observation rule, 22 bins spanning 22–80 °C remained, and 21 of 22 device differences retained the positive direction; the ten-observation rule retained only five bins at 76–80 °C and reversed the direction for all devices. Relative temperature slope was not retained, and complete-profile retention remained heterogeneous. The framework therefore provides an auditable basis for selecting endpoints and designing controlled retests.

The scope of these conclusions follows from the archived records themselves. The results establish cross-record structural persistence. Sensor life and remaining useful life require a documented time base, namely the duration preceding the aging-stage record and the interval before first retest, from which a rate of change per unit time is obtained. Degradation of sensitivity requires synchronized reference pressure at both stages, since sensitivity is the response of output to applied pressure. Retention of metrological characteristics requires traceable reference values together with a controlled loading path, which give accuracy, nonlinearity, hysteresis, and repeatability. Connecting this retention signature to calibration accuracy, qualification, or causal aging requires synchronized reference pressure, controlled pressure–temperature paths, defined exposures, and prospectively specified decision limits.

## Figures and Tables

**Table 1:** Test-procedure information carried by the archived primary-cohort records, and the measurements a confirmatory study would supply.

Procedure Attribute	In the Archived Records	Analysis Supported, or Measurement to Be Supplied
Carried by the archive and used in this study		
Temperature channel	16.125–82.8125 °C (aging stage); 11.5625–85.0625 °C (first retest)	Supports common-support conditioning on shared temperature bins
Pressure-channel output	6143–6144 valid pairs per record	Supports marginal and temperature-conditioned feature extraction
Device identifier	One-to-one across the two record groups	Supports paired device-level analysis (n=22)
Assembly status	Board-level records identified	Supports exclusion of records whose assembly status differs
To be supplied by a confirmatory study		
Storage or operating duration before the aging-stage record	—	Fixes the time base for a drift rate and for service life
Exposure temperature over the aging interval	—	Fixes the thermal dose, and converts the group label into a defined exposure
Exposure load or applied pressure over the aging interval	—	Fixes the mechanical dose, and allows a change to be attributed to it
Operating mode during exposure	Recorded for the repeat cohort only	Separates mode-dependent behavior from the record label
Elapsed interval before first retest	—	Separates short-term repeatability from change over time
Synchronized reference pressure	—	Places output on a metrological scale, giving accuracy, sensitivity, nonlinearity, and hysteresis
Row-level timestamps	—	Reconstructs loading sequence and equilibrium state
Loading program, path direction, and dwell	—	Separates hysteresis and path dependence from sensor response
Physical unit of the pressure-channel output	Unconfirmed	Permits numerical pooling across cohorts and comparison against a specification
Package, lot, and exposure history	—	Isolates batch and packaging effects

**Table 2:** Comparison axes and the inferential failure addressed by each axis.

Comparison Axis	Metric	Supported Inference	Failure Revealed
Between-device order	Spearman ρ and BCa interval	Whether the device hierarchy persists between records	Monotone ordering may survive a common level or scale shift
Identity-line agreement	CCC and paired difference	Whether feature values remain numerically close	High correlation can coexist with poor agreement
Common-temperature structure	*A*, *W*, β and $\Delta_{i}$	Whether relative output persists on shared temperature support	Raw differences may reflect unequal temperature occupancy
Complete response shape	Centered-profile correlation	Whether the relative trajectory is retained across temperature	Favorable scalar features can conceal local reversals

Notes: BCa, bias-corrected and accelerated; CCC, concordance correlation coefficient.

**Table 3:** Marginal-feature retention in the primary and disjoint same-mode repeat cohorts.

(A) Primary Aging-Stage to First-Retest Cohort (*n* = 22)						
			**Identity-Line Agreement**			
**Feature**	ρ **(BCa 95% CI)**	**FDR-** q	**CCC**	r	$C_{b}$	**Leave-One-Out** ρ **Range**
$Q_{0.05}$	0.886 [0.491, 0.983]	$2.10\times 10^{-7}$	0.968	0.973	0.995	0.869–0.970
$Q_{0.50}$	0.380 [−0.077, 0.700]	0.0808	0.048	0.682	0.071	0.287–0.510
$Q_{0.95}$	0.719 [0.351, 0.885]	$4.05\times 10^{-4}$	0.379	0.510	0.743	0.677–0.803
Standard deviation *S*	0.648 [0.328, 0.838]	0.00186	0.032	0.420	0.077	0.612–0.717
90% span $D_{90}$	0.614 [0.270, 0.832]	0.00296	0.154	0.264	0.583	0.568–0.679
(B) Disjoint Same-Mode Repeat Cohort (*n* = 16)						
**Feature**	ρ **(BCa 95% CI)**				**FDR-** q	
$Q_{0.05}$	0.624 [0.231, 0.860]				0.0164	
$Q_{0.50}$	−0.491 [−0.741, 0.030]				0.0667	
$Q_{0.95}$	0.756 [0.421, 0.925]				0.00353	
Standard deviation *S*	0.691 [0.130, 0.910]				0.00756	
90% span $D_{90}$	0.324 [−0.227, 0.708]				0.222	

Notes: The device is the resampling and deletion unit. BCa, bias-corrected and accelerated; CCC, concordance correlation coefficient; CI, confidence interval; FDR, false discovery rate. FDR-*q* is controlled separately over the five prespecified marginal features in each cohort. CCC is a descriptive identity-line concordance estimate; no equivalence margin was prespecified.

**Table 4:** The retention signature of the archived records, stated as the joint pattern across evidence levels. The signature is an ordinal description of which evidence each quantity supports; it is not a threshold-based classification, and no cut-off is applied to any column.

(A) Marginal Level, by Feature					
**Feature**	**Order Retained (Primary; Repeat)**	**Identity-Line Agreement,** $\mathrm{CCC}=r\;C_{b}$	**Factor Limiting Concordance**		**Evidence Supported**
$Q_{0.05}$	Yes; yes (0.886; 0.624)	High (0.968 = 0.973 × 0.995)	Neither factor limiting		Order and numerical agreement
$Q_{0.95}$	Yes; yes (0.719; 0.756)	Low (0.379 = 0.510 × 0.743)	Precision, r=0.510		Order only; association is weak
*S*	Yes; yes (0.648; 0.691)	Very low (0.032 = 0.420 × 0.077)	Accuracy, $C_{b}=0.077$ (u=4.84)		Order only; values systematically displaced
$D_{90}$	Yes; not recurrent (0.614; 0.324)	Low (0.154 = 0.264 × 0.583)	Precision, r=0.264		Neither transfers across cohorts
$Q_{0.50}$	Not supported; not supported (0.380; −0.491)	Very low (0.048 = 0.682 × 0.071)	Accuracy, $C_{b}=0.071$ (u=5.12)		Neither order nor agreement
(B) Temperature-Conditioned and Functional Levels, Cohort-Level					
**Quantity**		**Cross-Record Estimate**		**Evidence Supported**	
Relative offset *A*		ρ=0.496; BCa interval includes zero		Weak; persistent relative level not established	
Centered span *W*		ρ=0.491 [0.005, 0.794]		Weak; temperature-dependent spread partially ordered	
Relative temperature slope β		ρ=0.013 [−0.526, 0.558]		None; monotonic temperature component not retained	
Complete centered profile		Median within-device ρ=0.356; IQR 0.051–0.593; 17 of 22 positive		Heterogeneous; response shape not reproduced cohort-wide	
Conditional stage difference $\Delta_{i}$		Sign reverses between raw and common-bin support for all 22 devices		Direction is a property of the retained domain, not of the device	

Notes: The order-retained column reports whether the BCa interval excluded zero in the primary and disjoint repeat cohorts, with Spearman ρ in parentheses; full intervals and FDR-*q* values are given in Table 3. *u* is the location-shift term of Equation (4), expressed in units of the geometric mean standard deviation. The signature is read across a row: a single column describes one level of evidence, and features that agree on one level routinely diverge on another. The descriptors high, low, weak, and none are relative to the other entries in this table, since an acceptance meaning would require an equivalence margin that the uncalibrated output does not support.

**Table 5:** Observed retention patterns translated into requirements for a controlled retest protocol.

Observed Pattern	Implication for Retest Design	Required Controlled Measurement
$Q_{0.05}$ retained both device order and identity-line proximity; $Q_{0.95}$ and *S* retained order in two disjoint cohorts	Carry these features forward as candidate endpoints, but do not infer accuracy from rank retention alone, and nominate the endpoint prospectively	Traceable reference pressure at prospectively specified pressure–temperature states, with engineering tolerances defined before testing
Concordance was lost through the accuracy factor for *S* and $Q_{0.50}$ ($C_{b}=0.077$ and 0.071)	Treat the disagreement as a cohort-wide level displacement that a traceable reference could resolve, with device information retained	A traceable level reference at repeated fixed states, sufficient to estimate and, if justified, correct a common offset
Concordance was lost through the precision factor for $Q_{0.95}$ and $D_{90}$ (r=0.510 and 0.264)	Treat the disagreement as device-specific scatter that no offset correction removes	Replicated readings per device at fixed pressure and temperature levels, including reference-pressure uncertainty and repeatability
Raw and common-temperature stage differences had opposite signs; occupancy thresholds changed the retained domain	Standardize temperature coverage, dwell, and minimum support before comparing stage summaries	Time stamps, chamber and chip temperature where available, per-bin counts, dwell time, and a fixed thermal path
Complete profiles were heterogeneous and an upper-boundary excursion was stage dependent	Preserve loading direction and equilibrium status when response shape is an endpoint	Repeated ascending and descending pressure–temperature paths, loading order, a logged set-point program, and explicit equilibrium criteria

## Data Availability

The processed device-level feature tables, pseudonymized values underlying the additional figures, record-selection audit, and executable analysis workflows are provided in the Supplementary Materials. The underlying raw laboratory exports are not publicly available because they are proprietary test records. Requests may be directed to the corresponding author and require permission from the data owner. The archived data do not contain synchronized reference pressure or the missing test-procedure metadata identified in this article.

## Supplementary Materials

The following supporting information can be downloaded at https://www.mdpi.com/article/10.3390/s26175650/s1, Data S1, processed device-level features, robustness results, and record-selection audit; Data S2, pseudonymized values underlying Figure 3, Figure 4, Figure 7, Figure 10, and Figure 11; Code S1, executable inferential-analysis and primary-figure workflow; Code S2, executable additional-figure workflow; File S1, supplementary file description and reuse instructions.

## Abbreviations

The following abbreviations are used in this manuscript:

BCa	Bias-corrected and accelerated
CCC	Concordance correlation coefficient
CI	Confidence interval
CSV	Comma-separated value
FDR	False discovery rate
MEMS	Microelectromechanical systems
SHA-256	Secure Hash Algorithm 256-bit
